# Factors Associated With Fecal Microbiota Transplant Failure in the Treatment of Recurrent Clostridioides difficile Infection: A Single-Center Retrospective Study

**DOI:** 10.7759/cureus.45118

**Published:** 2023-09-12

**Authors:** Fatima Warraich, Syed H Sohail, Alexander Knee, Jacob Smith, Hans Schlecht, Daniel Skiest

**Affiliations:** 1 Internal Medicine, University of Massachusetts Chan Baystate Medical Center, Springfield, USA; 2 Office of Research/Epidemiology/Biostatistics Research Core, University of Massachusetts Chan Baystate Medical Center, Springfield, USA; 3 Infectious Disease, University of Massachusetts Chan Baystate Medical Center, Springfield, USA

**Keywords:** fmt failure, recurrent c diff, gut biome, fecal microbiota transplantation (fmt), clostridioides difficile infection

## Abstract

Background

*Clostridioides difficile *infection (CDI) is a major cause of hospital-acquired diarrhea and is associated with substantial morbidity and mortality. Recurrences following treatment are common. Fecal microbiota transplantation (FMT) is a therapeutic intervention in which stool from a healthy donor is administered to a patient with recurrent CDI. Studies to date of predictors of FMT failure have primarily included inpatients. In this study, we aimed to describe FMT failure rates within one year of FMT and evaluate factors associated with FMT failure.

Methodology

We conducted an exploratory retrospective study of consecutive patients who underwent outpatient FMT at a single tertiary care center in Western Massachusetts from December 2014 through September 2018. We collected patient data including demographics, CDI*-*related factors, and FMT-related factors. FMT failure was defined as non-response or recurrence of diarrhea, associated with positive stool *C. difficile* toxin or polymerase chain reaction. Unadjusted relative risk (RR) and 95% confidence intervals for factors associated with FMT failure were estimated using log-binomial regression.

Results

A total of 92 patients were included with a mean age of 64 years. CDI severity was mild or moderate in 73% and severe or fulminant in 27%. The most common FMT indication was recurrent CDI in 76% of patients. FMT failure occurred in 25 of 92 (27%) patients, with half occurring within 11 days. Factors associated with FMT failure were active malignancy (RR = 2.56), prior hospitalizations (RR = 2.42), and receipt of non-CDI antibiotics within six months of FMT (RR = 2.80). We did not observe strong associations for risk of FMT failure with age ≥65, sex, use of proton pump inhibitors or H2 receptor agonists, history of colectomy, immunosuppression, history of malignancy, diabetes, appendectomy, CDI severity, or probiotic use.

Conclusions

Active malignancy, prior CDI hospitalizations, and non-CDI antibiotics within six months before FMT were associated with FMT failure in the outpatient setting. Knowledge of the above factors may help inform shared decision-making with patients at risk for FMT failure.

## Introduction

*Clostridioides difficile* infection (CDI) is a major cause of hospital-acquired diarrhea and is associated with substantial morbidity and mortality [1]. Important risk factors for CDI include antibiotic therapy, advanced age, and hospital or nursing home stay [2]. The first-line management includes stopping the offending antibiotic if possible and treatment with antimicrobials active against CDI, usually vancomycin, metronidazole, or fidaxomicin [2]. Despite treatment with appropriate antibiotics, recurrences are not uncommon [3], occurring in 10-25% of patients. Among patients who have already experienced recurrent CDI (rCDI), subsequent recurrence occurs in up to 65% of patients [1,4].

Fecal microbiota transplantation (FMT) involves an infusion of stool from a healthy individual to a patient with presumed gut dysbiosis. The mechanism of action appears to be the establishment of a new gut microbiota community to restore normal gut function [5]. Multiple randomized controlled clinical trials have demonstrated the efficacy of FMT, with success rates ranging from 81% to 91%. FMT has become more available and acceptable in recent years leading to increased use for rCDI [5-8]. Treatment guidelines by the Infectious Diseases Society of America and the Society for Healthcare Epidemiology of America recommend FMT for patients with multiple recurrences of CDI, who have failed appropriate antibiotic treatments [9].

As noted, despite the relatively high efficacy of FMT for rCDI, FMT failure occurs in up to 9-19% of individuals. Delineating factors associated with failure of FMT in rCDI is important in choosing patients who are most likely to benefit from FMT versus other treatments for rCDI. A previous study identified the following factors that were associated with FMT failure: severe and fulminant disease, inpatient status at the time of FMT, and increased frequency of previous CDI-related hospitalizations [10]. In a 2022 meta-analysis of 20 observational studies, advanced age, severe CDI, inflammatory bowel disease (IBD), peri-FMT use of non-CDI antibiotics, prior CDI-related hospitalizations, inpatient status at the time of FMT, and poor quality of bowel preparation were predictors of FMT failure within two to three months [11].

While these studies have provided important information, they primarily included patients who were hospitalized at the time of FMT. Factors contributing to FMT failure in the outpatient population are not well defined and may differ from those described in hospitalized patients. In this study, our objective was to explore potential factors associated with FMT failure in the outpatient setting.

Some of the data in this paper were previously presented at the Annual Scientific Meeting of the American College of Gastroenterology in Charlotte, North Carolina, USA in October 2022 as a poster presentation.

## Materials and methods

We conducted a retrospective, exploratory, cohort study at a large tertiary care hospital in Western Massachusetts. Eligible patients were older than 18 years and had undergone outpatient FMT for CDI between December 1, 2014, and September 30, 2018. Patients were initially identified using our FMT data repository, which includes prospective data collection on all FMT procedures at our institution. Once eligibility was confirmed, subsequent data collection was managed using REDCap [12]. The Institutional Review Board waived the requirement for consent.

Measures collected prospectively (by the attending physicians overseeing the FMT procedures) from the FMT repository included demographics (age at the time of FMT, race, sex), comorbidities (IBD, diabetes mellitus, past or active malignancy, past or current radiation therapy), FMT characteristics (days from CDI relapse to initial FMT, use of non-CDI antibiotics in the prior six months, FMT route of delivery, proton pump inhibitor (PPI) use within 24 hours of FMT (if nasogastric administration), date of first FMT relapse).

Data subsequently collected from a review of the electronic medical record (EMR) included CDI history (history of colectomy, prior hospitalizations due to CDI), CDI severity (severe defined as CDI with the presence of leukocytosis (white blood cell count ≥15,000 cells/mL) or serum creatinine level >1.5 mg/dL) or fulminant (hypotension or shock, ileus, megacolon) [6]), FMT indication, medical history including prior intensive care unit (ICU) admission, immunosuppression (defined as a weakened immune system due to immunosuppressive medications, including any dose of glucocorticoid medication, organ transplant recipients, human immunodeficiency virus, diabetes, and inherited or primary immunodeficiencies), immunocompromised (active malignancy and/or met the criteria for immunosuppression above), prior appendectomy, probiotic use before FMT, bezlotoxumab use, use of PPIs and H2 receptor antagonists.

The main outcome of interest was FMT failure and time to failure, defined as the date of the first relapse recorded in the FMT repository. Relapse was identified based on follow-up by the attending physician as documented in the EMR by a clinic visit, follow-up phone call, or hospital visit. Follow-up began at the time of FMT and ended one year later at the date of relapse/reinfection or the date of the last visit in the hospital system (whichever came first). We were not able to distinguish relapse from reinfection. FMT failure was broadly defined as symptoms of CDI that did not resolve or patients with new symptoms consistent with CDI up to one year after FMT.

Due to the small, fixed sample size, we planned an exploratory, descriptive analysis evaluating the associations between baseline characteristics and FMT failure for up to one year. Categorical variables are presented as frequencies and percentages and stratified by FMT failure (stratified percentages estimate cumulative incidence). As this was a hypothesis-generating study, we used Fisher’s exact test and a conservative p-value <0.20 as suggestive of possible associations worth further investigation. To describe the stability of our estimates, we also calculated unadjusted relative risks (RRs) and 95% confidence intervals (CIs) using log-binomial regression. Analysis was conducted using Stata/MP version 17 (StataCorp, LP, College Station, TX, USA).

## Results

A total of 92 patients met the criteria for inclusion. The mean (standard deviation) age at FMT was 64 (19) years, and 78% were females (Table 1). The majority of the study population was white (87%). Significant medical comorbidities included diabetes (19%), active malignancy (21%), history of radiation therapy (10%), and IBD (7%).

**Table 1:** Univariable associations between patient characteristics and FMT failure within one year. ^1^: Fisher’s exact test. CDI = *Clostridioides difficile* infection; FMT = fecal microbiota transplant

		FMT failure within one year		
	Overall	No	Yes	
	n = 92, n (%)	n = 67 (72.8%), n (%)	n = 25 (27.2%), n (%)	P-value^1^
Patient demographics				
Age category at transplant				
<65 years	44 (47.8%)	31 (70.5%)	13 (29.5%)	0.647
≥65 years	48 (52.2%)	36 (75.0%)	12 (25.0%)	
Sex				
Male	20 (21.7%)	15 (75.0%)	5 (25.0%)	1.0
Female	72 (78.3%)	52 (72.2%)	20 (27.8%)	
Gastrointestinal history				
Prior hospitalization for CDI				
No	29 (31.5%)	25 (86.2%)	4 (13.8%)	0.076
Yes	63 (68.5%)	42 (66.7%)	21 (33.3%)	
Use of proton pump inhibitors or H2 receptor antagonists				
No	58 (63.0%)	42 (72.4%)	16 (27.6%)	1.0
Yes	34 (37.0%)	25 (73.5%)	9 (26.5%)	
History of prior colectomy				
No	82 (89.1%)	59 (72.0%)	23 (28.0%)	0.723
Yes	10 (10.9%)	8 (80.0%)	2 (20.0%)	
Other medical history				
Immunosuppressed status				
No	76 (82.6%)	57 (75.0%)	19 (25.0%)	0.358
Yes	16 (17.4%)	10 (62.5%)	6 (37.5%)	
Active malignancy				
No	73 (79.3%)	58 (79.5%)	15 (20.5%)	0.009
Yes	19 (20.7%)	9 (47.4%)	10 (52.6%)	
Diabetes mellitus				
No	75 (81.5%)	57 (76.0%)	18 (24.0%)	0.225
Yes	17 (18.5%)	10 (58.8%)	7 (41.2%)	
History of appendectomy				
No	77 (83.7%)	56 (72.7%)	21 (27.3%)	1.000
Yes	15 (16.3%)	11 (73.3%)	4 (26.7%)	
Antibiotics received within six months of FMT				
No	32 (34.8%)	28 (87.5%)	4 (12.5%)	0.027
Yes	60 (65.2%)	39 (65.0%)	21 (35.0%)	
FMT characteristics				
CDI severity				
Mild/Moderate	67 (72.8%)	51 (76.1%)	16 (23.9%)	0.415
Severe	15 (16.3%)	10 (66.7%)	5 (33.3%)	
Fulminant	10 (10.9%)	6 (60.0%)	4 (40.0%)	
Probiotic use before FMT				
No	62 (67.4%)	44 (71.0%)	18 (29.0%)	0.625
Yes	30 (32.6%)	23 (76.7%)	7 (23.3%)	

In relation to CDI history, nearly 69% of patients had a prior hospitalization related to CDI (median = 2, range = 1-5). The severity of CDI was mostly mild/moderate in 73%, severe in 16%, and fulminant in 11%. The majority of patients underwent FMT for rCDI (76%), followed by recurrent and severe CDI (19%), severe CDI (3%), and fulminant colitis (2%). A large majority of FMTs (95%) were given via the upper gastrointestinal (GI) route of delivery (46 nasogastric/orogastric, four G-tube/J-tube, eight esophagogastroduodenoscopies, and 29 capsules), while the remaining were given via the lower GI (two colonics, two ileostomies, and one colonoscopy).

A total of 25 (27.2%) patients had an FMT failure (refractory to treatment, relapse, or new CDI infection) within one year. Overall, 50% of failures occurred within 11 days. One patient had a failure within two months of FMT, but the date was not recorded. Among those without relapse, 67% were observed for one year while only 25% were observed for less than six months.

**Figure 1:**
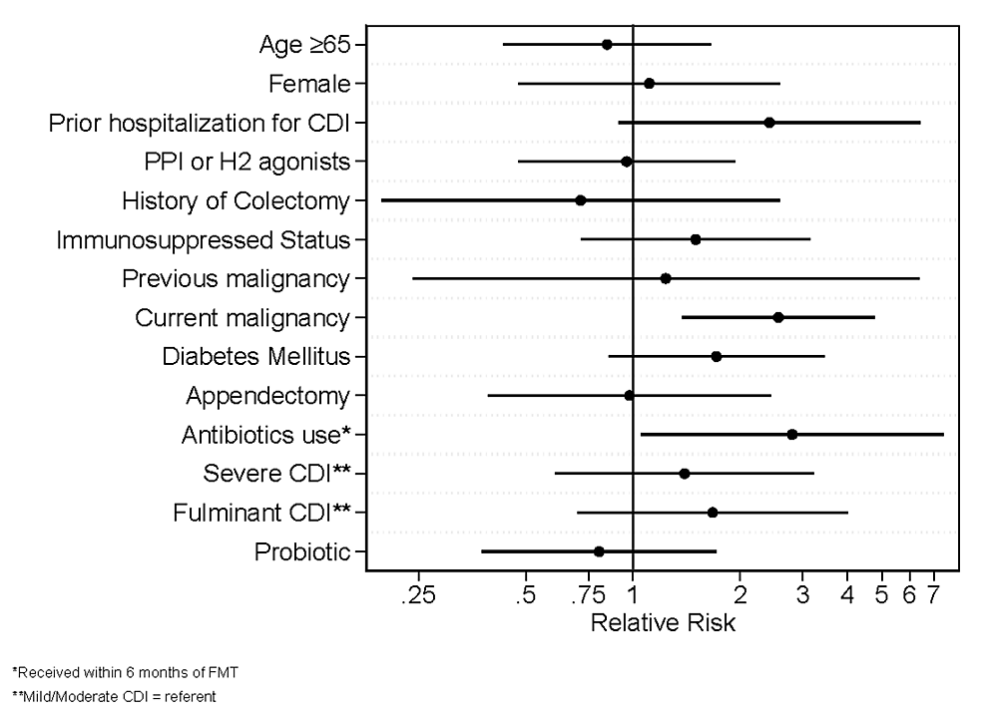
Relative risk. *: Received within six months of FMT; **: Mild/Moderate CDI = referent. CDI = *Clostridioides difficile* infection; FMT = fecal microbiota transplant; PPI = proton pump inhibitor

The following factors were associated with FMT failure in our study cohort: current or active malignancy (53% vs. 25%; RR = 2.56; 95% CI = 1.38-4.77), prior hospitalization for CDI (33% vs. 14%; RR = 2.42; 95% CI = 0.91-6.40), and receipt of non-CDI antibiotics within six months of FMT (35% vs. 13%; RR = 2.80; 95% CI = 1.05-7.46) (Figure 1). The most commonly prescribed antibiotics were carbapenems, penicillins, and third-generation cephalosporins. The following factors were not associated with FMT failure: age ≥65, sex, use of PPI or H2 receptor agonists, a history of colectomy, immunosuppression, prior malignancy, diabetes, a history of appendectomy, CDI severity, or probiotic use.

## Discussion

In our retrospective cohort study of patients who underwent FMT for CDI, therapy failed in 27%. We observed the following factors to be associated with FMT failure: active malignancy, prior CDI-related hospitalizations, and non-CDI antibiotic use within a six-month period before FMT. FMT failure often occurred soon after the procedure, with half of FMT failures occurring in fewer than two weeks.

A recent meta-analysis of 20 studies found the following risk factors for FMT failure: advanced age, severe CDI, IBD, peri-FMT use of non-CDI antibiotics, prior CDI-related hospitalizations, inpatient status, and poor quality of bowel preparation [11]. Another recent study found that early FMT failure (defined as within one month) was associated with severe CDI, fulminant CDI, inpatient FMT, and the number of hospitalizations for CDI before FMT [10]. Other studies have found a correlation between prior hospitalization and risk of rCDI [10]. Prior studies had a shorter time of follow-up and included both inpatient and outpatient FMT.

Our study was relatively small, only included outpatients, and followed patients for up to one year. This may be a potential reason for the difference in our results compared to other recent studies, which may have had increased power to determine contributing factors due to a larger sample size. It is also possible that patients included in the other studies, which included inpatients, may have worse overall health and higher rates of comorbidities in the inpatient cohort [10].

We observed that antibiotic use within a six-month period before FMT was associated with a 2.8-fold increased risk of FMT failure compared to those not receiving antibiotics. This finding is not unexpected as numerous prior studies have noted that antibiotic use is a major factor in the development of CDI. Infection occurs when there is disruption of the normal intestinal microbiota most often associated with antibiotic use, leading to an overgrowth of* C. difficile* [13].

Efforts to prevent CDI have included educating healthcare providers about the judicious use of antibiotics via antimicrobial stewardship programs, EMR alerts, and educational sessions for providers. At our facility, an EMR alert is shown when antibiotics are ordered for patients with recent CDI. In patients with rCDI, it may be advisable to also inform the prescribing clinician to consider that additional antibiotics may reduce the efficacy of FMT for rCDI if needed in the future.

In our study, immunosuppression at the time of FMT was associated with a nearly three-fold increase in the risk for FMT failure. The traditional selection of FMT candidates excludes or discourages the use of FMT in immunocompromised patients due to concerns about an increased risk of adverse events, including bacteremia due to translocation and limited efficacy data [14]. However, a few recent observational studies have suggested the benefit of FMT for rCDI in immunocompromised patients with few adverse effects. Cheng et al. described a cohort of 94 patients with a history of solid organ transplants who underwent FMT for CDI. A total of 58% had a primary cure rate which was defined as the resolution of diarrhea or negative polymerase chain reaction test after a single FMT. In addition, 91% of the patients were eventually cured, which could have involved multiple FMT and CDI antibiotics [15]. In our cohort, 54% of immunocompromised patients did not have a documented recurrence after FMT. Additional studies are needed to determine the safety and efficacy of FMT for rCDI in this population.

We also observed that active malignancy was associated with a 2.5-fold increase in the risk of FMT failure. This is likely because patients with cancer may be immunocompromised either due to receipt of therapy that suppresses the immune response, or a weak host response due to the malignancy, and/or comorbid conditions associated with immune suppression [16]. Additionally, these patients may have more contact with healthcare facilities resulting in more opportunities for colonization with *C. difficile*, which can lead to an increased risk of rCDI [16].

In contrast to our finding of an increased risk for rCDI in patients with active cancer, a few recent small studies have observed FMT to be largely successful in patients with cancer. Ali et al. reported on 19 patients with malignancies who underwent FMT for CDI and had an 84% success rate for FMT [17]. The patients in the study who failed FMT were noted to suffer from chemotherapy complications and required more antibiotic use. Similarly, Mendelsohn et al. studied 10 patients with solid tumors undergoing chemotherapy who received FMT for CDI, eight of whom were cured [17]. Further studies should consider stratifying based on the type of malignancy.

Our study is limited by the small sample size, retrospective nature, and the fact that we could not distinguish between treatment failure and re-infection. Moreover, data on the receipt of antibiotic use was incomplete as we only recorded inpatient antibiotic use at our facility. While we used a p-value of 0.20 to suggest possible associations, a lack of statistical power may result in missing possible risk factors. While not all participants without relapse were observed for the full year, a large majority were. We explored time-to-event analyses; however, due to the rarity of events, estimates were unstable. We assumed that patients with less than one year of observation time would have presented to our facility which is the largest tertiary care center in the area and the only facility conducting FMT in Western Massachusetts. This information may be of use to future researchers studying outpatient FMT.

## Conclusions

Multiple modifiable and non-modifiable risk factors are known to be associated with FMT failure. Our study adds to the data on the understanding of FMT failure and its associated risk factors in the outpatient setting. The use of antibiotics, active malignancy, and prior hospitalization appear to be important risk factors contributing to FMT failure and warrant additional investigation. This information is valuable as physicians can use the data in discussions with patients to engage in shared decision-making with patients regarding the risk of failure. Additionally, this information can help physicians determine suitable candidates for FMT.
